# Neutrophil/Lymphocyte ratio has no predictive or prognostic value in breast cancer patients undergoing preoperative systemic therapy

**DOI:** 10.1186/s12885-015-2005-3

**Published:** 2015-12-29

**Authors:** Christoph Suppan, Vesna Bjelic-Radisic, Marlen La Garde, Andrea Groselj-Strele, Katharina Eberhard, Hellmut Samonigg, Hans Loibner, Nadia Dandachi, Marija Balic

**Affiliations:** 1 Division of Oncology, Department of Internal Medicine, Medical University of Graz, Auenbruggerplatz 15, 8036 Graz, Austria; 2 Department of Gynaecology and Obstetrics, Medical University of Graz, Auenbruggerplatz 14, 8036 Graz, Austria; 3 Research Facility for Biostatistics, Center for Medical Research, Medical University of Graz, Stiftingtalstraße 24, 8010 Graz, Austria; 4 Apeiron Biologics AG, Campus-Vienna-Biocenter 5, 1030 Vienna, Austria

**Keywords:** Neutrophil-to-lymphocyte-ratio, Breast cancer, Preoperative, Predictive marker, Prognostic marker, Inflammation

## Abstract

**Background:**

The primary goal of preoperative systemic treatment (PST) in patients with breast cancer is downsizing of tumors to enhance the rate of breast conserving surgery. Additionally, preoperative systemic treatment offers the possibility to assess for chemosensitivity of early stage disease. In various cancers the prognostic value of neutrophil/lymphocyte ratio (NLR) was demonstrated, indicating that high NLR determines worse prognosis of the patients. The goal of our study was to evaluate the predictive and prognostic value of NLR in early stage breast cancer patients undergoing PST.

**Methods:**

247 female patients with histologically proven breast cancer were analysed in this retrospective analysis. The NLR before the initiation of PST was documented. Histopathological response in surgically removed specimens was evaluated using a modified Sinn regression score and the pCR defined as no invasive tumor in primary tumor and lymph nodes. NLR was correlated with response to PST and disease free survival.

**Results:**

PST was categorized into five groups (anthracycline containing, anthracycline and taxane containing, taxane containing, hormone treatment and other chemotherapies). pCR rate was defined as no invasive rest of tumor either in primary tumor or (ypT0 = Sinn) or in primary tumor and in lymph nodes (ypT0isypN0). Median NLR in patients without any invasive tumor rest was significantly higher than in patients either with some invasive tumor rest or not responding to chemotherapy. Despite this primary difference, the results were not stable across the analysed treatment groups particularly in the group with highest pCR rates (taxane and anthracycline treatment). Further, no association with disease free survival could be observed.

**Conclusions:**

Although there was a reverse trend with the higher NLR prior to systemic treatment in patients who achieved pCR, we could not demonstrate predictive or prognostic value of NLR in the cohort of early stage breast cancer patients treated with PST.

## Background

Breast cancer is the most commonly diagnosed cancer in women worldwide and the third leading cause of cancer-related death in Europe and the United States [1].

Preoperative systemic treatment (PST) has become increasingly accepted as a treatment option for primary breast cancer, not only for locally advanced or inflammatory disease, but also for lower stage breast cancer [2].

The main goal of PST is downsizing of the tumor in order to enhance the rate of breast conserving surgery. The greatest advantage of PST is that the treatment success can be monitored, thus providing an opportunity for novel drugs to be evaluated at an early stage of disease. Moreover, the assessment of tumor response to chemotherapy provides prognostic information for ongoing patient management with the pathological complete response (pCR) as a surrogate marker for survival for most intrinsic subtypes of breast cancer. Preoperative setting may provide the opportunity to change the therapy in case of clinical progress early in the course of treatment [3].

Despite enormous progress in molecular classification of breast cancer, most important factors determining patients’ prognosis are still pathological stage including the size of primary tumor and lymph node involvement, expression of recognized biomarkers like hormone receptors and Her2neu and measurement of proliferation. According to the Sankt Gallen International Expert Consensus, classification of intrinsic subtypes of breast cancer may be performed according to pathological parameters ER, PR, Her2neu and Ki67 [4]. Besides molecular characteristics of cancer, it is becoming increasingly clear that also host-related factors, such as nutritional and functional status and immunological response are associated with clinical outcome. During the last decades it was repeatedly shown that host inflammatory response plays a crucial role in carcinogenesis and disease progression [5]. For example, the systemic inflammatory response with elevated C-reactive protein (CRP) or serum amyloid A was reported to be associated with reduced survival, as demonstrated for several tumors like metastatic prostate, gastrooesophageal, colorectal, pancreatic or breast cancer [6].

One reason may be that tumor growth can cause tissue inflammation and hence increased CRP levels. Furthermore there is also evidence that cancer cells can increase the production of inflammatory proteins [6]. Another possibility is that an elevated CRP might reflect an immune response to tumor antigens. Chronic inflammation, finally, might play an aetiological role in cancer by creating a microenvironment enriched with reactive oxygen and nitrogen species released by inflammatory cells, thereby causing DNA alterations. Therefore, tumor growth may be promoted by inflammatory cytokines and proteins.

In a similar way to the modified systemic inflammation-based Glasgow Prognostic Score (mGPS), that uses thresholds of CRP and albumin and has been found to correlate with worse prognosis of cancer patients, the value of haematological components and of the differential white cell count was investigated for its prognostic and predictive value [5, 7]. Patients with a high density of lymphocytes in the stroma of tumors, for instance, had a better clinical outcome compared to patients with a low density of lymphocytic infiltration of tumor stroma. In contrast, high density of neutrophils was associated with poor clinical outcome [8–10]. Neutrophil/lymphocyte ratio (NLR) is an easily available marker of the systemic inflammatory response. The ratio consists of the absolute neutrophil and absolute lymphocyte blood cell counts.

Since the role of NLR in early stage breast cancer patients is largely unknown, the aim of our study was to evaluate the predictive and prognostic value of NLR in patients selected for treatment with PST at our hospital.

## Methods

This retrospective study was performed in patients consecutively selected for PST at the Medical University of Graz between 2001 and 2012. Data from 298 patients with histologically verified breast cancer were included. Patients were treated either at the Division of Oncology, Department of Internal Medicine or at the Department of Gynaecology and Obstetrics. After tumor resection, all patients were included into a follow-up-programme of one of the institutions. These follow-up-examinations normally included clinical visits with blood analysis, radiological analyses (Computed tomography or X-ray, ultrasound, mammography) and gynaecological investigations. Initial follow up evaluations were performed every 3 months within the first 3 years, 6 months in the 4^th^ and 5^th^ year, and thereafter annually. Clinicopathological data were documented in medical and pathological records.

PST was categorised into the following five groups: Anthracycline containing, taxane containing, anthracycline and taxane containing, hormone treatment and other chemotherapies. Laboratory data on complete blood count, including absolute cell counts of neutrophils and lymphocytes before the first cycle of PST were documented. The classification of breast cancer was evaluated by using the TNM-system according to the UICC-classification of 2002. The histologic response to chemotherapy was classified into different regression grades using a modified Sinn regression score from 0 to 4 (0 = no effect, 1 = resorption and tumor sclerosis, 2 = minimal residual invasive tumor, 3 = residual noninvasive tumor only, 4 = no tumor detectable) [11]. Pathological complete response (pCR) was defined as no invasive tumor rest (score 3 and 4), and as response to therapy we defined score 1–4 vs. score 0 without any therapeutic effect. In addition, we have performed an analysis according to the definition of pCR as no invasive tumor rest in the primary tumor and lymph nodes (ypT0isypN0 or Sinn score 3 and 4 and ypNO) [12].

Since the laboratory values were available only for 247 out of 298 patients, 247 patients were further included in analyses and the NLR was calculated. After comparison of the common disease characteristics there was no difference within patients with available NLR values (*n* = 247) and the entire patient cohort (*n* = 298). Therefore all subsequent statistical analyses were performed with the cohort of patients with NLR values (*n* = 247). The median follow-up time for the 247 patients was 123 months (CI 95 % 115.6-120.4). The NLR was correlated with response to PST and disease-free survival (DFS). Disease free survival (DFS) was defined as the time in months from the date of biopsy to the first documented recurrence during the follow up or the date of the final documentation. The aim of the current study was to investigate the prognostic and predictive value of pretreatment NLR in breast cancer patients selected for PST at our Institution. The study has been approved by the local ethical committee of the Medical University of Graz (EK: 25–233 ex 12/13). Signed informed consent forms were not required from the participants.

### Statistical analyses

All statistical analyses were performed using SPSS (version 20.0) software (SPSS Inc., Chicago, IL, USA).

Univariable and multivariable Cox proportional hazards model, including tumor grade, receptor status, clinical tumor size, lymph node involvement and NLR were fit to determine the clinicopathological parameters that were significantly statistically associated with DFS and the results were reported, including hazard ratios (HR) and 95 % confidence intervals (CI). Binary logistic regression analyses were performed to assess the influence of NLR on complete pathological and therapy response. Odds Ratios (OR) estimated from logistic regression were reported with corresponding 95 % confidence intervals (95 % CI). A *p* < 0.05 was considered statistically significant.

## Results

Invasive ductal carcinoma (IDC) was diagnosed in 78.5 %, invasive lobular carcinoma in 10.9 % and mixed histological types in 8.5 % of patients. The median age of the patients was 52 years (range 28 – 78). According to the menopausal status, 44.9 % of patients were premenopausal, 51.0 % of patients were postmenopausal and 2.0 % perimenopausal.

Clinical tumor size before PST was classified as cT1 in 5.7 %, cT2 in 49.8 %, cT3 in 27.5 % and cT4 in 10.9 %. Grade 1 tumors were found in 6.9 %, grade 2 in 34.8 % and grade 3 in 49.4 %. Lymph node status was negative in 39.7 % patients and positive in 59.5 % patients.

ER positive tumors were found in 60.7 %, ER negative in 38.5 %, whereas PR positive tumors were diagnosed in 54.3 % of patients, PR negative in 42.9 %. The Her2 expression was negative in 66.4 % and positive in 19.8 % patients.

Anthracycline-containing chemotherapy was given in 32.8 % patients, anthracycline and taxane containing chemotherapy in 58.3 %, only taxane containing therapy in 2.8 % patients and 6.1 % patients received other chemotherapies. The median number of cycles was 4.0 (range 1–8).

pCR according to Sinn score 3 and 4 was found in 29 (11.7 %) patients. In 30.4 % of patients there was no response to chemotherapy (Sinn score 0) and in remaining 69.6 % patients at least some response was observed (Sinn score 1–4). pCR including the lymph node status (ypT0is ypN0) was found in 21 patients (8.5 %). All patients’ characteristics are summarized in Table 1.

**Table 1:** Baseline characteristics *n* = 247

Parameter	Number	Percent
Age		
Median (range)	52 (28–78)	
Menopausal Status		
Premenopausal	111	44.9
Perimenopausal	5	2.0
Postmenopausal	126	51.0
Unknown	5	2.0
Tumor Size		
cT 1	14	5.7
cT 2	123	49.8
cT 3	68	27.5
cT 4	27	10.9
Unknown	15	6.1
Histology		
ILC	27	10.9
IDC	194	78.5
Mixed	21	8.5
Others	2	0.8
Unknown	3	1.2
Tumor Grading		
G1	17	6.9
G2	86	34.8
G3	122	49.4
Unknown	22	8.9
Her2 Status		
Negative	164	66.4
Positive	49	19.8
Unknown	34	13.8
ER Status		
Negative	95	38.5
Positive	150	60.7
Unknown	2	0.8
PR Status		
Negative	106	42.9
Positive	134	54.3
Unknown	7	2.8
Lymph node Status		
Negative	98	39.7
Positive	147	59.5
Unknown	2	0.8
Neoadjuvant Therapy		
Anthracycline	81	32.8
Anthracycline/Taxane	144	58.3
Taxane	7	2.8
Others	15	6.1
No of therapy cycles		
Median (range)	4 (1–8)	
Response to Therapy		
No (Sinn 0)	75	30.4
Yes (Sinn 1–4)	172	69.6
pCR		
No (Sinn Score 0,1,2)	218	88.3
Yes (Sinn Score 3,4)	29	11.7
pCR		
No (all others)	216	87.4
Yes (Sinn 3 or 4 and pN0)	21	8.5
Unknown	10	4.0
Recurrence		
No	150	60.7
Yes	95	38.5
Unknown	2	0.8

Abbreviation: *pCR* pathologic complete remission

The median NLR of patients with response to therapy (Sinn score 1–4) was 4.97 (range 0.58-25.67) and in patients without response (Sinn score 0) 2.63 (range 0.55-24.80). In the univariate logistic regression model of NLR with regard to response to therapy there was a significant correlation between NLR and response (*p* = 0.012). However, the R Square with 0.04 indicated that the NLR is only a weak predictive parameter for response to therapy in our patients (see Table 2).

**Table 2:** Univariable logistic regression model of Neutrophil-lymphocyte ratio with regard to response to therapy (*n* = 247)

Risk factor	β	Odds ratio (95 % CI)	*p*
Neutrophil-lymphocyte ratio (NLR)	0.087	1.091 (1.019-1.167)	0.012

Abbreviations: *CI* confidence interval, *β* regression coefficient, response to therapy = Sinn score 1–4

Patients with pCR according to Sinn score 3 and 4 (*n* = 29) had a median NLR of 6.92 (range 1.29-25.67), and patients who did not achieve pCR (*n* = 218) had a median NLR of 3.85 (range 0.55-24.80). Although there seems to be a trend (*p* = 0.051) towards better response in patients with higher NLR this trend was not statistically significant with an R Square of 0.028. Therefore, no multivariate analyses were calculated. Using pCR according to ypT0is ypN0 as the outcome variable similar results were achieved (*p* = 0.053 and R Square of 0.032). This data were summarized in Tables 3 and 4.

**Table 3:** Univariable logistic regression model of Neutrophil-lymphocyte ratio with regard to complete pathological response (*n* = 247)

Risk factor	β	Odds ratio (95 % CI)	*p*
Neutrophil-lymphocyte ratio (NLR)	0.071	1.073 (1.000-1.152)	0.051

Abbreviations: *CI* confidence interval, *β* regression coefficient, complete pathological response = Sinn score 3 and 4

**Table 4:** Univariable logistic regression model of Neutrophil-lymphocyte ratio with regard to complete pathological response (ypT0isypN0) (*n* = 247)

Risk factor	β	Odds ratio (95 % CI)	*p*
Neutrophil-lymphocyte ratio (NLR)	0.077	1.081 (0.999-1.169)	0.053

Abbreviations: *CI* confidence interval, *β* regression coefficient

An additional analysis was made with respect to the different intrinsic subtypes. Even in different intrinsic subtypes there was no group where we could identify NLR as a predictive parameter for pCR. Values according to subtypes were as follows: for triple negative tumors (*n* = 49) *p* = 0.296; R Square = 0.033, ER or PR positive tumors (*n* = 112) *p* = 0.818; R Square = 0.002 and Her2-positive tumors (*n* = 49) *p* = 0.108; R Square = 0.090. These results were summarised in Table 5.

**Table 5:** Univariable logistic regression model of Neutrophil-lymphocyte ratio with regard to intrinsic subtypes (*n* = 247)

Risk factor	Number	β	Odds ratio (95 % CI)	*p*
Neutrophil-lymphocyte ratio (NLR)				
Triple negative	49	0.072	1.075 (0.938-1.232)	0.296
ER or PR positive	112	−0.024	0.976 (0.796-1.197)	0.818
Her2 positive	49	0.185	1.203 (0.960-1.507)	0.108
Unknown	37	0.088	1.092 (0.971-1.228)	0.142

Abbreviations: *CI* confidence interval, *β* regression coeeficient

Due to the heterogeneity of PST and significant correlation of pCR with the combination of taxanes and anthracyclines we further analysed a subgroup of patients who received taxane and anthracycline containing treatment for predictive value of NLR (*n* = 144). Out of 144 patients, 23 patients in this group achieved pCR (16 %). In these patients with pCR the median NLR was 6.92 (range 0.55-24.80), and was lower than in patients with no pCR (*n* = 121) with a median NLR of 7.54 (range 1.29-23.17). Even in this homogenously treated group of patients there was no predictive value of NLR for pCR.

The median disease-free survival time was 66 months (range 3–207 months). In the further analyses of the association of NLR with DFS, there was no statistical significance between the NLR and DFS (*p* = 0.363, HR 0.978; 95 % CI 0.932-1.026). However, univariable Cox regression analyses of disease-free survival showed a prognostic significance for the variables tumor grade, progesterone receptor status, clinical tumor size, and lymph node status. In the multivariate analyses there was no significant association between tumor grade and DFS. Receptor status, tumor size and lymph node involvement remained statistically significant also in the multivariate analyses. Similar to the univariate analysis, there was no association between NLR and DFS in the multivariate analyses (*p* = 0.738, HR 1.009, 95 % CI 0.957-1.064). These results are summarized in Table 6.

**Table 6:** Univariable and multivariable Cox proportional analyses of clinico-pathological parameters with regard to disease free survival

Parameter			Univariable analyses		Multivariable analyses	
	No recurrence	Recurrence	HR (95 % CI)	*p* value	HR (95 % CI)	*p* value
Tumor grade				0.041		0.353
G1 + G2	69	32	1.00		1.00	
G3	70	52	1.534 (1.017-2.314)		1.278 (0.762-2.142)	
PR (biopsy)				0.034		0.002
Negative	58	46	1.00		1.00	
Positive	91	43	0.655 (0.443-0.968)		0.448 (0.268-0.748)	
cT pretreatment				0.001		0.002
cT1 + cT2	100	37	1.00		1.00	
cT3 + cT4	44	49	1.955 (1.309-2.919)		2.244 (1.342-3.750)	
pN (tumor)						
pN0	72	19	1.00		1.00	
pN1	60	52	3.255 (1.929-5.492)	<0.001	2.813 (1.440-5.496)	<0.002
pN2 + pN3	15	18	4.003 (2.057-7.788)	<0.001	6.793 (3.070-15.030)	<0.001
NLR	150	95	0.978 (0.932-1.026)	0.363	1.009 (0.957-1.064)	0.738

Abbreviations: *CI* confidence interval, *cT* clinical tumor size, *NLR* neutrophil lymphocyte ratio, *HR* hazard ratio, *PR* progesterone receptor

To summarize, although there seemed to be a trend to higher NLR prior to PST in patients who achieved pCR, we could not show an association between the preoperative NLR and DFS or response to chemotherapy in our 247 patients.

## Discussion

The predictive and prognostic value of NLR has been increasingly investigated and implicated for its prognostic and predictive value [7]. In the present retrospective study we show that in early stage breast cancer patients selected for PST, elevated NLR does not predict response to PST nor does it correlate with the prognosis of these patients.

Many groups have investigated the prognostic value of NLR in different tumor types and stages of disease. NLR was demonstrated as independently prognostic in patients with upper gastrointestinal malignancy, more robustly at later stage cancers [7]. In three studies that investigated 470 patients with hepatocellular carcinoma receiving neoadjuvant chemotherapy, an elevated NLR was significantly associated with increased risk of recurrence and risk of death and was an independent predictor of DFS and OS [13–15]. Further studies on 547 patients with oesophageal cancer receiving neoadjuvant chemotherapy reported an association between elevated NLR and poor prognosis. Sato et al., 2012 could show an independent association between NLR and pathological response to treatment, while in the study of Sharaiha et al., 2011 no association with response to treatment was found [16, 17]. Wang et al., 2013 investigated the predictive value of the NLR in patients with cervical cancer undergoing neoadjuvant chemotherapy before radical hysterectomy and could not confirm this parameter as a significant predictor for survival. Short follow-up and different biological behaviour of cancer seemed to be two reasons explaining the variance in their results compared to other studies [18].

Further studies have demonstrated the prognostic significance of NLR including Absenger et al., 2013 in early stage colon cancer, Stotz et al., 2014 in stage III colon cancer, Pichler et al., 2013 in renal cancer patients and Szkandera et al., 2013b in pancreatic cancer [19–22].

A study of over 400 breast cancer patients evaluating the prognostic factor of the NLR, showed that patients with a higher NLR were older, had more lymph node involvement and metastases. The pretreatment NLR was an independent, significant predictor of long-term mortality [23]. However, in this study patient data were collected prior to initiating of any therapy including radiotherapy, chemotherapy or surgery. Another study that analysed pretreatment NLR values of over 400 breast cancer patients in Korea concluded that patients with an elevated NLR showed poorer disease-specific survival than patients with lower NLR [24].

A retrospective analysis of 1,527 patients with breast cancer has been published analysing the preoperative NLR and its predictive or prognostic relevance. Moreover they calculated the derived N/L Ratio (dNLR) including leukocyte counts in order to compare which parameter is better in terms of predicting prognosis in patients with breast cancer [25]. Disease-free survival and overall survival were both significantly associated with the calculated cut-off values of NLR and dNLR. In multivariate analyses they showed that high NLR is an independent prognostic factor for OS and DFS, while prognostic significance could not be shown for high dNLR.

Krenn-Pilko et al., 2014 evaluated the effect of preoperative platelet-to-lymphocyte Ratio (PLR) on cancer-specific survival, overall survival and distant metastases-free survival in a total of 793 patients and showed that a high PLR is a consistent factor for poor prognosis in breast cancer patients, while an elevated NLR was only significantly associated with cancer-specific survival in univariable analysis and a significant impact was not detected in multivariable analysis [26].

In our study only patients undergoing PST were included. Pathological response including pCR has a known impact on prognosis in case of pathological complete response, which is a suitable surrogate end point for patients with HER2-positive (nonluminal), Triple-negative, and luminal B/HER2-negative tumors but not for luminal B/HER2-positive and luminal A tumors [27].

Outcome of patients with breast cancer in the neoadjuvant setting, where downsizing of the tumor is the major aim of treatment and patients present at an early stage without distant metastasis has different prognosis from other tumor types in a more advanced stage of disease with more aggressive tumor behaviour. Furthermore, until recently, breast cancer was not regarded as immunogenic tumor. There was no correlation of immune suppression with higher incidence of disease, however, outcome of immunosuppressed patients was shown to be worse [28, 29]. Increasing amount of data has now demonstrated that both in triple negative neoadjuvant breast cancer and Her2 positive breast cancer, lymphocytic infiltration in primary tumors had predictive value, with patients with high levels of lymphocytic infiltration in primary tumors generating greater benefit of neoadjuvant treatment and more frequently achieving pathological complete response [30].

Prognostic value of lymphocytic infiltration at primary diagnosis in adjuvantly treated triple negative patients was demonstrated by Loi et al., 2014, and recently confirmed in independent analyses. It has been even suggested that benefit from neoadjuvant Trastuzumab is highest in patients with high levels of lymphocytic infiltration [31].

There are several potential reasons why our results did not demonstrate predictive or prognostic value in our patient study. Our cohort of preoperatively treated patients consisted of patients with all three intrinsic subtypes, luminal cancers, Her2 positive cancers and triple negative breast cancers. So far, no studies have demonstrated higher levels of lymphocytic infiltration in hormone receptor positive tumors and their prognostic value. This may explain our results. Furthermore, local lymphocytic infiltration at the primary tumor site is unlikely to be reflected in systemic blood count. Finally, previous studies demonstrated that an elevated NLR was not only associated with poor survival in cancer patients, it was also a negative prognostic predictor for cardiovascular events [32], an independent marker of mortality in patients with bacteraemia [33] and a factor for worse prognosis in patients with chronic kidney disease [34]. NLR measurement can be affected by all these factors as well as by hepatic or renal dysfunction and abnormal thyroid function tests. These studies showed that NLR alone without any other inflammatory markers is insufficient to provide enough information to clinicians as a predictive or prognostic marker, which has also been mentioned by Absenger et al., 2013 and Balta et al., 2013 [20, 35].

## Conclusions

Although haematological components of the systemic inflammatory response and especially the NLR may have clinical utility in predicting survival in different cancers, our retrospective study failed to show an impact of NLR on disease-free survival or therapy response in breast cancer patients undergoing PST and thus does not represent a robust predictive or prognostic factor in this patient cohort. Due to the heterogeneity of breast cancer and different influencing factors of NLR measurement, the role of systemic inflammation parameters in breast cancer in neoadjuvant situation should be further evaluated and be differentiated from other cancer types.

## Abbreviations

DFS: disease-free survival
ER: estrogen receptor
PR: progesterone receptor
TNM: classification of malignant tumors (Tumor/Nodes/Metastasis)
UICC: Union Internationale Contre le Cancer
